# Outcomes in Patients Requiring Veno‐Venous Extracorporeal Membrane Oxygenation After Cardiac Surgery: An Analysis From the PELS‐1 Study

**DOI:** 10.1111/aor.70093

**Published:** 2026-01-11

**Authors:** Pasquale Nardelli, Silvia Mariani, Maria Elena De Piero, Bas C. T. van Bussel, Michele Di Mauro, Ann‐Kristin Schaefer, Diyar Saeed, Matteo Pozzi, Luca Botta, Udo Boeken, Robertas Samalavicius, Karl Bounader, Xiaotong Hou, Jeroen J. H. Bunge, Hergen Buscher, Leonardo Salazar, Bart Meyns, Michael A. Mazzeffi, Marco L. Sacha Matteucci, Sandro Sponga, Kollengode Ramanathan, Claudio Russo, Francesco Formica, Pranya Sakiyalak, Antonio Fiore, Daniele Camboni, Giuseppe Maria Raffa, Rodrigo Diaz, I‐wen Wang, Jae‐Seung Jung, Jan Belohlavek, Vin Pellegrino, Giacomo Bianchi, Matteo Pettinari, Alessandro Barbone, José P. Garcia, Kiran Shekar, Glenn Whitman, Roberto Lorusso, PELS Investigators

**Affiliations:** ^1^ Cardiovascular Research Institute Maastricht (CARIM) Maastricht University Maastricht the Netherlands; ^2^ Cardiac Critical Care Unit IRCCS San Raffaele Scientific Institute Milan Italy; ^3^ Cardiac Surgery Unit Fondazione IRCCS San Gerardo dei Tintori Monza Italy; ^4^ Department of Intensive Care Medicine Maastricht University Medical Center Maastricht the Netherlands; ^5^ Care and Public Health Research Institute Maastricht University Maastricht the Netherlands; ^6^ Department of Cardiac Surgery Medical University of Vienna Vienna Austria; ^7^ Heart Center Niederrhein Helios Hospital Krefeld Krefeld Germany; ^8^ Department of Cardiac Surgery Louis Pradel Cardiologic Hospital Lyon France; ^9^ Division of Cardiac Surgery IRCCS Azienda Ospedaliero‐Universitaria di Bologna Bologna Italy; ^10^ Department of Cardiac Surgery, Medical Faculty Heinrich Heine University Duesseldorf Germany; ^11^ Department of Anesthesiology, Centre of Anesthesia, Intensive Care and Pain Management Vilnius University Hospital Santariskiu Klinikos Vilnius Lithuania; ^12^ Division of Cardiothoracic and Vascular Surgery Pontchaillou University Hospital Rennes France; ^13^ Center for Cardiac Intensive Care, Beijing Institute of Heart, Lung, and Blood Vessels Diseases, Beijing Anzhen Hospital Capital Medical University Beijing China; ^14^ Department of Intensive Care Adults Erasmus MC Rotterdam the Netherlands; ^15^ Deparment of Cardiology, Thoraxcenter Erasmus MC Rotterdam the Netherlands; ^16^ Department of Intensive Care Medicine Center of Applied Medical Research, St Vincent's Hospital, Darlinghurs, NSW and University of New South Wales Sidney Australia; ^17^ Department of Cardiology Fundación Cardiovascular de Colombia Bucaramanga Colombia; ^18^ Department of Cardiovascular Sciences University of Leuven Leuven Belgium; ^19^ Department of Anesthesiology University of Virginia Charlottesville USA; ^20^ SOD Cardiochirurgia Ospedali Riuniti ‘Umberto I—Lancisi—Salesi’ Università Politecnica delle Marche Ancona Italy; ^21^ Division of Cardiac Surgery, Cardiothoracic Department University Hospital of Udine Udine Italy; ^22^ Cardiothoracic Intensive Care Unit, National University Heart Centre National University Hospital Singapore Singapore; ^23^ Cardiac Surgery Unit, Cardiac Thoracic and Vascular Department Niguarda Hospital Milan Italy; ^24^ Cardiac Surgery Clinic, University of Salento, Department of Experimental Medicine, Vito Fazzi Hospital Lecce Italy; ^25^ Division of Cardiovascular and Thoracic Surgery, Department of Surgery, Faculty of Medicine Siriraj Hospital Mahidol University Bangkok Thailand; ^26^ Department of Cardio‐Thoracic Surgery University Hospital Henri‐Mondor, Créteil Paris France; ^27^ Department of Cardiothoracic Surgery University Medical Center Regensburg Regensburg Germany; ^28^ Department for the Treatment and Study of Cardiothoracic Diseases and Cardiothoracic Transplantation IRCCS‐ISMETT (Istituto Mediterraneo per i Trapianti e Terapie ad Alta Specializzazione) Palermo Italy; ^29^ Cardiac Surgery Unit, Department of Precision Medicine in Medical Surgical and Critical Area (Me.Pre.C.C.) University of Palermo Palermo Italy; ^30^ ECMO Unit, Departamento de Anestesia Clínica Las Condes Santiago Chile; ^31^ Division of Cardiac Surgery Memorial Healthcare System Hollywood Florida USA; ^32^ Department of Thoracic and Cardiovascular Surgery Korea University Anam Hospital Seoul South Korea; ^33^ 2nd Department of Internal Medicine, Cardiovascular Medicine General Teaching Hospital and 1st Faculty of Medicine Charles University in Prague Prague Czech Republic; ^34^ Intensive Care Unit The Alfred Hospital Melbourne Victoria Australia; ^35^ Ospedale del Cuore Fondazione Toscana “G Monasterio” Massa Italy; ^36^ Department of Cardiovascular Surgery Ziekenhuis Oost‐Limburg Genk Belgium; ^37^ Cardiac Surgery Unit IRCCS Humanitas Research Hospital Rozzano Milan Italy; ^38^ IU Health Advanced Heart & Lung Care Indiana University Methodist Hospital Indianapolis Indiana USA; ^39^ Adult Intensive Care Services, The Prince Charles Hospital and the University of Queensland Brisbane Queensland Australia; ^40^ Cardiac Intensive Care Unit Johns Hopkins Hospital Baltimore Maryland USA; ^41^ Cardio‐Thoracic Surgery Department Maastricht University Medical Centre Maastricht the Netherlands

**Keywords:** acute respiratory failure, cardiac surgery, extracorporeal life support, mechanical respiratory support, postcardiotomy

## Abstract

**Background:**

Acute respiratory failure after cardiac surgery is an uncommon complication, affecting morbidity and mortality. In these patients, respiratory extracorporeal membrane oxygenation (ECMO) support may be beneficial, as it may help reduce pulmonary vasoconstriction and the impact of respiratory pressures on the heart. Nevertheless, literature reports of postcardiotomy veno‐venous (V‐V) ECMO use are sporadic.

**Methods:**

This retrospective, multicenter cohort study analyzes data from the PELS‐1 registry, focusing on adult patients who required V‐V ECMO following cardiac surgery. PELS‐1 was conducted across 34 cardiac surgery centers in 16 countries from 2000 to 2020.

**Results:**

The study included 24 patients who received V‐V ECMO over a total of 2163 patients requiring postocardiotomy extracorporeal support (1.1%). The median age was 64[50–69] years, and 16/24 (67%) were male. Median Euroscore II was 6.2[3.1–19.6]. Most patients required prolonged cardiopulmonary bypass (CPB) time (208[110–350] min). V‐V ECMO was initiated in the ICU in 21 patients (87.5%) after a median of 5 [2–12] days postoperatively. ECMO support rapidly normalized gas exchange and lactate levels. However, complications were frequent: bleeding (10/22, 45.5%), acute kidney injury (10/24, 41.7%), pneumonia (10/24, 41.7%), and arrhythmias (7/24, 29.2%) were the most frequent ones. In‐hospital mortality was high, with only 21.7% discharged alive. One‐year survival was 12.5%.

**Conclusions:**

Reported outcomes of patients receiving V‐V ECMO after cardiac surgery are poor, despite effective correction of gas exchange. Early recognition of isolated respiratory failure and careful patient selection should be promoted. Further research is needed to optimize management in this high‐risk population.

## Graphical abstract

We report an insight from the PELS‐1– a multicentre, international registry focusing on postocardiotomy extracorporeal support use between 2000 and 2020. We focused on 24/2163 (1.1%) patients requiring V‐V ECMO after cardiac surgery. Mortality was high, with 21.7% of patients discharged alive and an overall 1‐year survival probability of 12.5%.

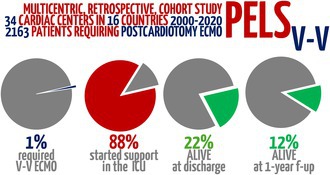

## 1 Introduction

Acute respiratory failure (ARF) following cardiac surgery is a serious complication, affecting 5%–10% of cardiac surgery operations and significantly contributing to morbidity and mortality [1, 2]. On top of “classic” causes of postoperative respiratory failure—including ventilator‐associated pneumonia and aspiration pneumonia—the postcardiotomy setting carries some peculiar conditions, notably related to the underlying cardiac disease and the extent of surgical intervention. While pulmonary edema resulting from left ventricular dysfunction is a well‐recognized cause of cardiogenic respiratory failure, right ventricular mechanisms may be based on positive fluid balance and central venous congestion [3]. As a part of a vicious cycle, venous congestion of the splanchnic circulation promotes gut edema and bacterial translocation, which can amplify systemic inflammatory responses and contribute to secondary pulmonary injury [4]. Systemic inflammation, fluid overload, and high transfusion rate may be additional major players contributing to postoperative ARF [5]. Exposure of blood to artificial surfaces and shear stress triggers the release of pro‐inflammatory cytokines, leading to increased pulmonary vascular permeability, interstitial and alveolar edema, and impaired gas exchange [6]. Extracorporeal circulation can also serve as a “first hit,” priming the lungs for further injury from secondary insults such as infection or ventilator‐induced lung injury [7]. Finally, prolonged suspension of mechanical ventilation during cardiopulmonary bypass may contribute to the pathophysiology of ARF after cardiac surgery—with negative effects arising from alveolar collapse and systemic inflammatory response [8]. In this multifaceted scenario, preexisting comorbidities, including older age, obesity, and preexisting lung conditions or preoperative events, can contribute to precipitate this delicate balance.

After cardiac surgery, veno‐venous extracorporeal membrane oxygenation (V‐V ECMO) may be a viable option in patients suffering refractory compromised gas exchange despite maximal ventilatory support [9]. The initiation of respiratory ECMO support notably reduces pulmonary vasoconstriction and allows lung protective ventilation, thus reducing the impact of respiratory pressures on the right ventricle [10]. These mechanisms may result in positive hemodynamic effects and in a reduction of inotropic support [11]. However, postcardiotomy patients pose specific challenges, including difficulties related to cannulation in a recently operated chest [12], the need for ECMO‐related anticoagulation with high bleeding risk, and the necessity of careful patient selection, as concomitant cardiac dysfunction may require veno‐arterial (V‐A) or hybrid ECMO configurations [13]. Consequently, only a few and limited case‐series‐related reports of V‐V ECMO use after cardiac surgery exist in the literature [14, 15, 16, 17].

The present analysis of the Postcardiotomy Extracorporeal Life Support (PELS) [18] aims to describe baseline characteristics and clinical outcomes of patients receiving V‐V ECMO support after cardiac surgery. We hypothesize that patients requiring V‐V ECMO after cardiac surgery represent a distinct high‐risk subgroup, experiencing high mortality rates in spite of effective correction of gas exchange compromise and facilitation of lung protective ventilation.

## 2 Materials and Methods

### 2.1 Study Design and Population

The PELS‐1 Study is a multicenter, observational, retrospective study collecting clinical information on patients who received extracorporeal life support following cardiac surgery in 34 centers from 16 countries between January 2000 and December 2020. Details from the study have been previously described (ClinicalTrials.gov: NCT03857217) [18, 19, 20, 21]. For the current study, all patients receiving postcardiotomy V‐V ECMO support within the mentioned time frame were eligible for inclusion. Patients with missing information on survival or ECMO initiated for non‐cardiac surgical indications were excluded.

### 2.2 Data Collection

Data were collected by trained personnel at each participating institution and included demographics, comorbidities, cardiac surgical details, ECMO parameters, laboratory values, complications, and outcomes (Supporting Information). Data were stored using a dedicated electronic case report form (data.castoredc.com, Castor, Amsterdam, The Netherlands), according to predefined variable and outcome definitions (Supporting Information). The full dataset was centrally managed by the coordinating center. The primary outcome was in‐hospital mortality, further differentiated between on ECMO mortality (if the patient died while on ECMO support) and mortality after weaning (if the patient died after ECMO separation but before hospital discharge).

### 2.3 Statistical Analysis

Continuous variables are presented as median with 1st and 3rd quartile. Categorical variables are expressed as frequencies and percentages. Survival analysis was performed using Kaplan–Meier curves. Analysis of continuous variables over time was performed by applying the two‐way analysis of variance for repeated measures. A *p* value < 0.05 was considered significant. All data were merged from de‐identified files into SPSS 26.0 (IBM, Armonk, NY), and R 4.1.2 (R Foundation for Statistical Computing, Vienna, Austria) for data management and statistical analysis.

### 2.4 Ethical Considerations

All PELS‐1 investigations are conducted in accordance with institutional ethical standards and the 1975 Declaration of Helsinki. Primary institutional review board (IRB) approval was obtained at the leading center (Maastricht University Medical Centre+, Maastricht, The Netherlands, IRB‐approval METC‐2018‐0788, February 27, 2019). Need for informed consent was waived based on the retrospective nature of the study, the emergency of the performed procedures, and the pseudonymization of shared data. IRB approval was obtained in all centers based on the leading center's protocol.

## 3 Results

In total, 2163 patients were included in the PELS‐1 Study. After excluding patients who missed primary outcome data (*n* = 72), underwent V‐A ECMO (*n* = 2058), right ventricular support with oxygenator (*n* = 3), or uncertain ECMO configurations (*n* = 6), a total of 24 patients receiving V‐V ECMO were considered for the present investigation (Supplementary Figure 1).

**FIGURE 1:**
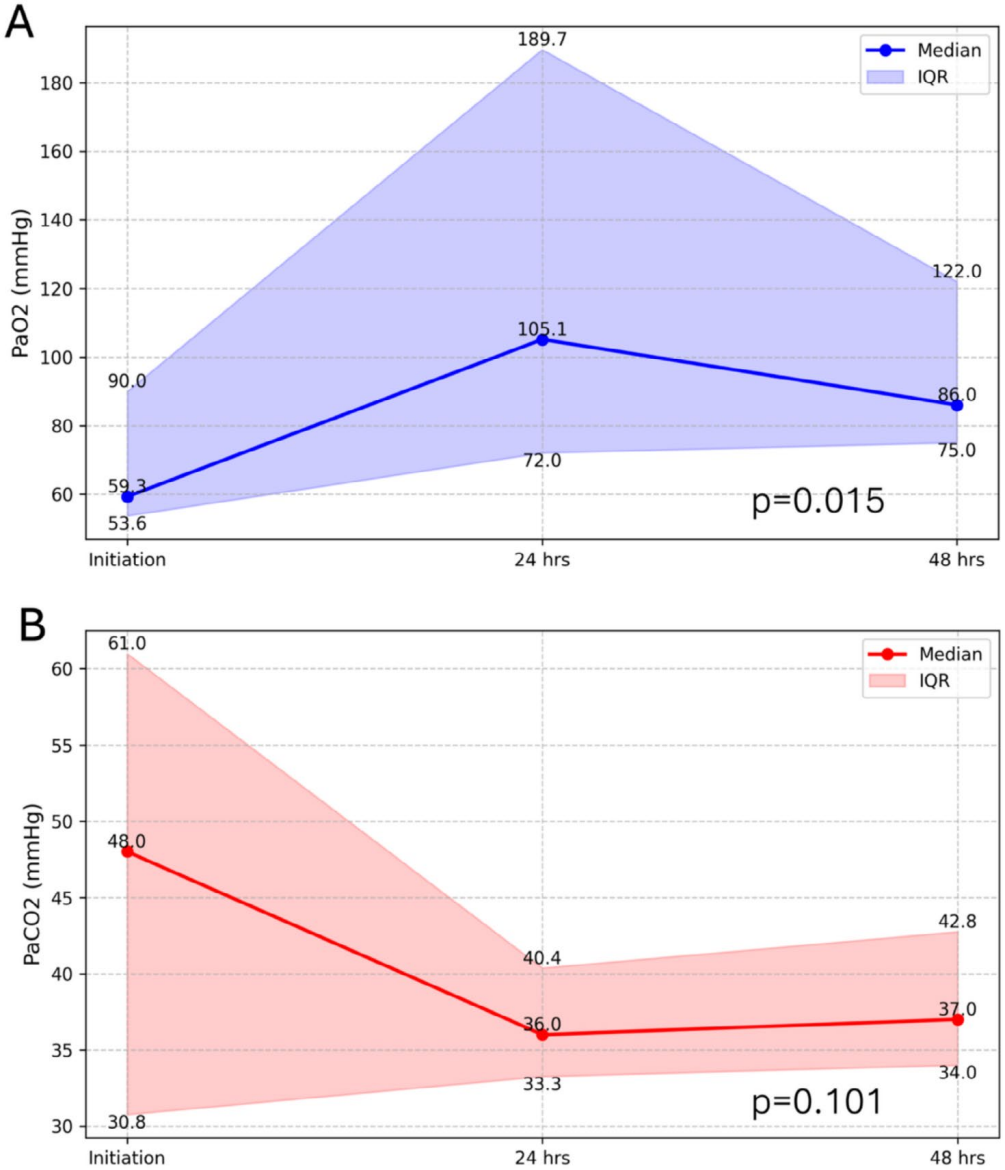
Partial pressure of oxygen (Panel A) and partial pressure of carbon dioxide (Panel B) trends over time in patients requiring postcardiotomy V‐V ECMO. [Color figure can be viewed at wileyonlinelibrary.com]

**FIGURE 2:**
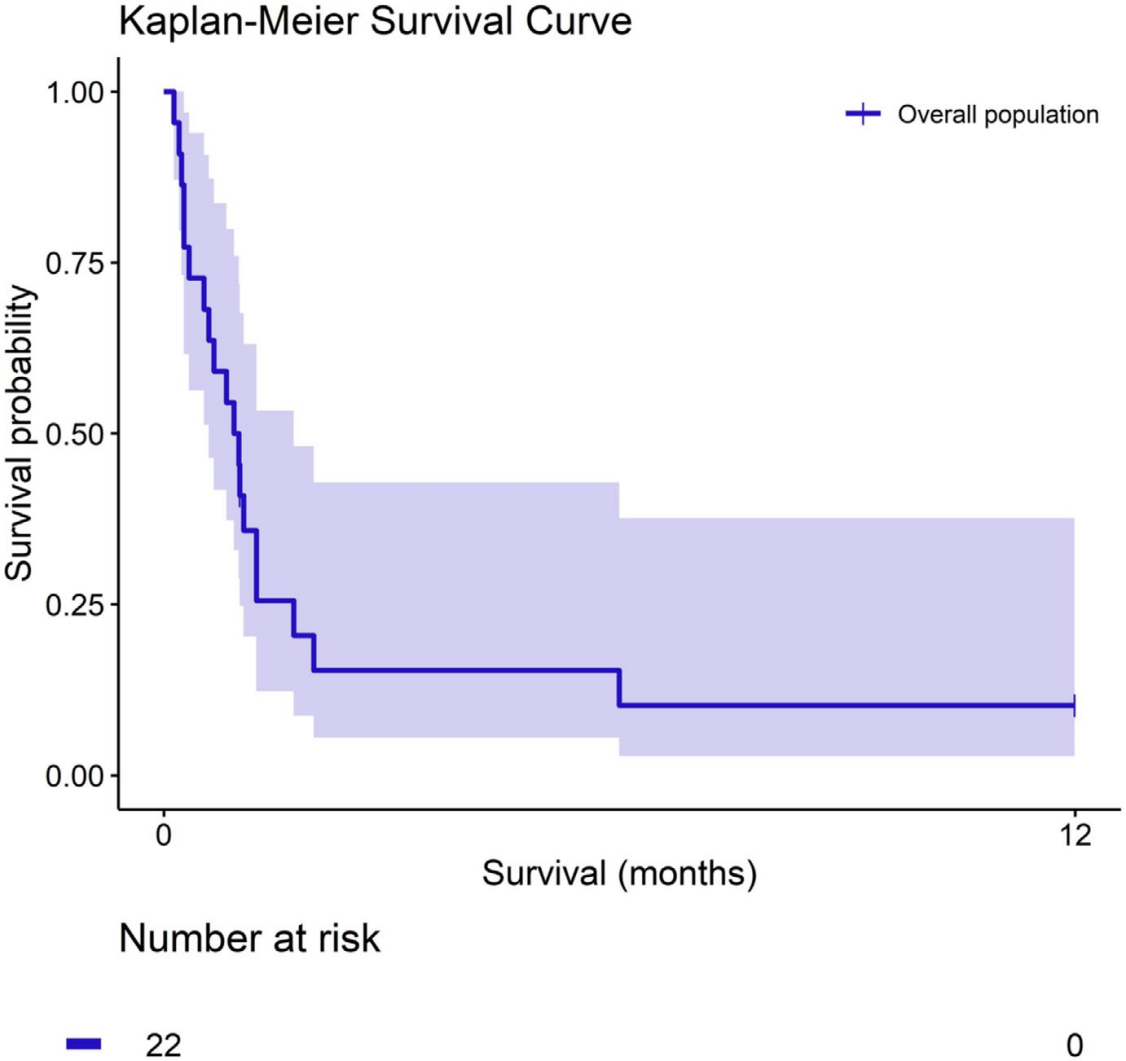
Kaplan‐Meyer of mortality of patients requiring postcardiotomy V‐V ECMO. [Color figure can be viewed at wileyonlinelibrary.com]

The cohort had a median age of 64 [50–69] years, with 8/24 (33.3%) female patients and a median body mass index of 27 [23.2–29.0] kg/m^2^. The most prevalent comorbidities included hypertension (70.8%), atrial fibrillation (29.2%), and diabetes (20.8%; Table 1). Preoperatively, patients had a median left ventricular ejection fraction of 55% [50–62] and a median EuroSCORE II of 6.2 [3.1–19.6]. The most common surgical procedures were aortic valve surgery in 13/24 (54.2%), surgery involving the ascending aorta in 11/24 (45.8%), and mitral valve surgery in 7/24 (29.1%), with 19/24 (79.2%) patients undergoing isolated procedures (Table S1). Patients underwent a median cardiopulmonary bypass (CPB) time of 208 [110–350] minutes and a median aortic cross‐clamp time of 114 [56–200] minutes (Table S1). Most patients (87.5%) were cannulated for ECMO in the intensive care unit after surgery, at a median time of 5 [2–12] days after surgery (Table 2). Partial pressure of oxygen increased and normalized after ECMO initiation (*p* = 0.015, Figure 1A) and partial pressure of carbon dioxide showed a trend in reduction and normalization during the first 48 h of support (*p* = 0.101, Figure 1B). Lactate levels also showed a progressive trend in reduction in these patients (*p* = 0.165, Figure S2). The median ECMO support duration was 163 [59–328] hours, with femoro‐femoral cannulation being the most common configuration (58.3%). Two patients (8.3%) required conversion to V‐A and one (4.1%) to veno‐arterovenous (V‐AV) ECMO. Postoperative complications were frequent, including bleeding (10/22, 45.5%), acute kidney injury (10/24, 41.7%), pneumonia (10/24, 41.7%), and arrhythmias (7/24, 29.2%). Mortality was high, with only 21.7% of patients discharged alive. Fifteen patients died on ECMO (83.3%) after a median support time of 90.5 [35–297] hours (Table 3). One‐year follow‐up was completed in all patients, with an overall 1‐year survival probability of 12.5% (Figure 2).

**TABLE 1:** Preoperative characteristics of patients requiring V‐V ECMO.

	V‐V ECMO patients	Data availability
	*n* = 24	
Demographics		
Age (years)	64 [50–69]	24 (100%)
Weight (kg)	72 [60–91]	24 (100%)
Height (m)	1.68 [1.64–1.77]	24 (100%)
Body mass index (kg/m^2^)	27.0 [23.2–29.0]	24 (100%)
Body surface area (m^2^)	1.79 [1.64–2.07]	24 (100%)
Female sex	8 (33.3%)	24 (100%)
Race		24 (100%)
White	16 (66.7%)	
Asian	4 (16.7%)	
Hispanic	3 (12.5%)	
Unknown	1 (4.2%)	
Black	0	
Comorbidities		
Hypertension	17 (70.8%)	24 (100%)
Dialysis	1 (4.2%)	24 (100%)
Previous myocardial infarction	2 (8.3%)	24 (100%)
Previous endocarditis	1 (4.2%)	24 (100%)
Smoking	4 (16.7%)	24 (100%)
Previous stroke	1 (4.2%)	24 (100%)
Atrial fibrillation	7 (29.2%)	24 (100%)
Diabetes mellitus	5 (20.8%)	24 (100%)
Chronic obstructive pulmonary disease	2 (8.3%)	24 (100%)
Peripheral vascular disease	1 (4.2%)	24 (100%)
Preoperative characteristics		
Left ventricular ejection fraction (%)	55 [50–62]	24 (100%)
Euroscore II	6.2 [3.1–19.6]	24 (100%)
Previous cardiac surgery	5 (20.8%)	24 (100%)
NYHA class III/IV	11 (45.8%)	24 (100%)
Preoperative IABP	2 (8.3%)	24 (100%)
Preoperative mechanical ventilation	4 (16.7%)	24 (100%)
Preoperative cardiogenic shock	3 (12.5%)	24 (100%)
Emergent and urgent surgery	11 (45.8%)	24 (100%)
Preoperative acute pulmonary oedema	2 (8.3%)	24 (100%)
Preoperative vasopressors	2 (8.3%)	24 (100%)
Center characteristics		
Heart transplantation centers	9 (37.5%)	24 (100%)
LVAD‐only centers	9 (37.5%)	24 (100%)
Non‐heart transplantation, non‐LVAD centers	6 (25%)	24 (100%)

*Note:* Continuous variables are presented as median with 1st and 3rd quartile. Categorical variables are expressed as frequencies and percentages.

Abbreviations: IABP, intra‐aortic balloon pump; LVAD, left ventricular assist device; NYHA, New York Heart Association.

**TABLE 2:** ECMO indications and characteristics.

	V‐V ECMO patients	Data availability
	*n* = 24	
ECMO indication		20 (83%)
Failed CPB separation for respiratory compromise	1 (4.5%)	
Pulmonary hemorrhage	1 (4.5%)	
Postoperative respiratory failure	18 (81.8%)	
ECMO implant timing		24 (100%)
Intra‐operative	3 (12.5%)	
Intensive care unit	21 (87.5%)	
ECMO postoperative implant time (day)	5 [2–12]	24 (100%)
ECMO duration (hours)	163 [59–328]	24 (100%)
Cannulation site		24 (100%)
Right atrium + femoral vein	3 (12.5%)	
Femoro‐femoral	14 (58.3%)	
Femoro‐jugular	7 (29.1%)	
Necessity to switch to V‐A ECMO	2 (8.3%)	24 (100%)
Necessity to switch to V‐AV ECMO	1 (4.1%)	24 (100%)

*Note:* Continuous variables are presented as median with 1st and 3rd quartile. Categorical variables are expressed as frequencies and percentages.

Abbreviations: CPB, cardiopulmonary bypass; ECMO, extracorporeal membrane oxygenation; V‐A, veno‐arterial; V‐AV, veno‐arterial venous.

**TABLE 3:** Postoperative complications and outcomes of patients requiring V‐V ECMO.

	V‐V ECMO patients	Data availability
	*n* = 24	
Postoperative bleeding	10 (45.5%)	22 (92%)
Thoracic, requiring rethoracotomy	5 (22.7%)	
Cannulation site	3 (13.6%)	
Cerebral hemorrhage	2 (9.1%)	
Acute kidney injury	10 (41.7%)	24 (100%)
Pneumonia	10 (41.7%)	24 (100%)
Arrhythmias	7 (29.2%)	24 (100%)
Bowel ischemia	5 (20.8%)	24 (100%)
Septic shock	4 (16.7%)	24 (100%)
Necessity of vascular surgery	3 (12.5%)	24 (100%)
Necessity of abdominal surgery	1 (4.2%)	24 (100%)
Right ventricular failure	0	24 (100%)
ICU stay (days)	18.5 [7–37]	24 (100%)
Mechanical ventilation time (hours)	15.0 [7–30]	24 (100%)
Hospital stay (days)	25.0 [10–37]	24 (100%)
Left ventricular ejection fraction at discharge (%)	50.0 [45–50]	24 (100%)
Mortality	19 (79.2%)	24 (100%)
Death timing		23 (96%)
Deceased after ECMO separation	3 (16.7%)	
Deceased on ECMO	15 (83.3%)	

*Note:* Continuous variables are presented as median with 1st and 3rd quartile. Categorical variables are expressed as frequencies and percentages.

Abbreviations: ECMO, extracorporeal membrane oxygenation; ICU, intensive care unit.

## 4 Discussion

This multicenter analysis from the PELS study represents one of the largest contemporary series of postcardiotomy patients requiring V‐V ECMO. Our findings confirm that postcardiotomy V‐V ECMO is rarely used in 1.1% of cardiac surgery patients requiring extracorporeal support. It is mainly applied in patients with a high‐risk pre‐ and intra‐operative profile including pre‐operative intubated patients, redo surgeries, urgent/emergency operations including valves or ascending aorta procedures. V‐V ECMO initiation is predominantly (87.5%) postoperative, occurring 5 days after surgery. Despite the initial normalization of hypoxemia, 12.4% of patients require conversion to circulatory support. Postcardiotomy V‐V ECMO patients show a high in‐hospital mortality (78.3%) mainly observed during support (83.3%).

Survival data require careful considerations, as reported mortality for V‐V ECMO exceeds that of V‐A ECMO (60.5%) in the postcardiotomy population described by PELS study [21]—differently than what happens in the general population. This finding possibly reflects the peculiarities of cardiac surgery, a setting in which V‐A ECMO is more familiar and generally considered earlier, with V‐V ECMO mostly used as a bailout solution. Moreover, reported mortality for postcardiotomy V‐V ECMO is also substantially higher than that of “classic” pulmonary ECMO patients, with latest reports from ELSO describing a 59% survival over 63 876 patients [22]. This further reflects the fact that V‐V ECMO after cardiac surgery is considered a rescue strategy, and applied late in patients already suffering multiorgan dysfunction and without standardized inclusion and exclusion criteria.

Patients requiring postoperative V‐V ECMO are consistent with typical cardiac surgery patients in terms of age and sex. However, the high prevalence of comorbidities hints at a vulnerable population with a reduced pre‐operative physiological reserve or an unstable condition. The median EuroSCORE II of 6.2 confirms the elevated preoperative risk profile of these patients. Moreover, we report long CPB and cross‐clamp times in the studied population, possibly due to surgical complexity as valves and ascending aorta procedures were common in the analyzed population. Extended duration of extracorporeal circulation and aortic clamping are recognized risk factors for pulmonary dysfunction after cardiac surgery, through mechanisms of inflammatory response, pulmonary endothelial damage, intraoperative fluid overload, high transfusion rate, and ischemia–reperfusion injury [23, 24].

Despite the pre‐ and intra‐operative severe risk profile for lung damage, most V‐V ECMO cannulations happened in the ICU, with a median postoperative time to ECMO of 5 days, possibly indicating that respiratory failure in these patients was progressive, hinting at postoperative pneumonia, or that V‐V ECMO initiation was delayed in favor of more conservative initial approaches. However, timing of ECMO initiation may be a key contributor in explaining excess mortality in our cohort. As a comparison, the study by Hafen et al. included 50% of patients who received V‐V ECMO within 24 h of the index operation, and earlier initiation was associated with better outcomes [14]. While the optimal timing of V‐V ECMO initiation in postcardiotomy patients remains controversial and a matter of investigation, it is possible that earlier extracorporeal treatment could improve outcomes in this peculiar setting as already demonstrated for V‐A ECMO [21].

Our data demonstrates that V‐V ECMO can provide rapid and effective correction of arterial blood gas abnormalities, within a short time frame after initiation of support (Figure 1), and may also contribute to revert tissue hypoxemia, as lactate levels tended to decrease progressively. However, these factors did not translate into improved survival, possibly because of the high incidence of non‐pulmonary complications.

The rate of conversion from V‐V to V‐A or V‐AV ECMO of 12.5%, even if similar to that reported in previous literature [16, 17], is also worth some additional considerations. These clinical events entail two possible scenarios: either the primary respiratory failure evolved with hemodynamic collapse or more subtle cardiogenic shock phenotypes were overlooked at initial evaluation. As isolated severe respiratory failure after cardiac surgery is a rare entity, efforts must be taken to correctly identify it and support it with the correct extracorporeal therapy. The use of a pulmonary artery catheter may help to identify postcardiotomy heart failure in a timely manner. A large series suggests that the use of a pulmonary artery catheter in cardiac surgery was associated with a reduced incidence of postoperative respiratory failure and overall improved survival [25].

Reports of V‐V ECMO use after cardiac surgery were summarized in Table 4. This scenario is rarely described, with small single‐center series. All authors reported very high mortality rates, between 36% and 46% in smaller series [16, 17] and 62% in larger ones [14, 15]. The extremely high mortality observed in this study requires careful evaluation of the selection criteria of patients who might be eligible for V‐V ECMO after cardiac surgery and identification of optimal ECMO initiation timing. While the small cohort of the present study does not permit identifying independent predictors of mortality, previous reports suggest that the severity scores (such as RESP or APACHE II) may accurately predict outcomes. Song et al. [17] reported a RESP risk class IV or V in all non‐survivors. Also, age may play an important role on outcomes, with survivors being significantly older than non‐survivors in Nakamura et al. [16] report. Based on literature data, few strategies may help improve outcomes in this delicate population undergoing such a modality of extracorporeal support. Early initiation of extracorporeal support [26], a better prevention of complications—with particular attention to bleeding [27] and infections [28]—and the use of hybrid configurations of ECMO may all be considered to reduce postcardiotomy ECMO morbidity. Accordingly, veno‐arterovenous ECMO may offer advantages for patients with respiratory failure and some degree of cardiac dysfunction, while minimizing the risk of differential hypoxemia associated with peripheral V‐A ECMO [29]. Further research should focus on this peculiar strategy to define the best extracorporeal support strategy for postcardiotomy patients with varying degrees of cardiac and respiratory dysfunction.

**TABLE 4:** Previous studies dealing with post cardiotomy V‐V ECMO.

Author, year	Study population	Age	Time to ECMO	Survival to discharge	Major complications
PELS‐1, 2025	24	64 [50–69]	5 days [2–12]	21.7%	Bleeding (45%), AKI (42%), pneumonia (42%)
Hafen, 2021	22	60 ± 15	50% < 24 h, rest median 4 days	38%	Not detailed
Song, 2016	13	Not specified	7.5 days (non‐HTx)	54%	Sepsis, bleeding, multi‐organ failure
Nakamura, 2013	11	63 ± 17	Not specified	64%	Bleeding, ischemic colitis, heart failure
Takagaki, 2019	8	79 ± 9	Not specified	37.5%	Bleeding, DIC, thrombosis

Our study has several limitations. First, the retrospective nature of the registry limits causal inferences. Second, the small sample size reflects the infrequency of V‐V ECMO use following cardiac surgery but limits the possibility of identifying mortality predictors. Third, due to the nature of the PELS registry, we lack detailed information on ancillary strategies, including ventilator settings and weaning protocols, which may influence outcomes. Also, no severity score was assessed before ECMO initiation. Fourth, the extended time frame of the registry and its multicentric nature may imply variability in technology and treatment protocols. The low incidence of V‐V ECMO use in the PELS registry, which only represented 1.1% of the total 2163 patients requiring postcardiotomy extracorporeal support, may reflect the rarity of isolated respiratory failure after cardiac surgery. However, the present study reports a substantially higher mortality than other single center series [16, 17]. The included patients had a high pre‐operative risk profile with a high Euroscore II; thus, they might have been prone to the observed negative outcomes regardless of V‐V ECMO initiation. Moreover, a proportion of these patients may have experienced subtle phenotypes of postcardiotomy shock, leading to late recognition and partly explaining the rate of switch to arterial modalities and the excess mortality. Unfortunately, no additional data about why V‐V ECMO was preferred over V‐A were collected. Nevertheless, the present study presents one of the largest series of patients requiring respiratory ECMO support after cardiac surgery and provides valuable insights into the use of this peculiar strategy. Severe unexplained respiratory failure after cardiac surgery requires further attention from the scientific community, as guidelines and structured algorithms are required to avoid misdiagnosis of postcardiotomy shock and to guide the prompt initiation of a tailored extracorporeal support. Clinical protocols involving pulmonary artery catheterization may be considered to optimize diagnosis and treatment in this specific subset of patients.

## 5 Conclusions

Respiratory failure requiring V‐V ECMO after cardiac surgery is uncommon, yet characterized by a high complexity, a high rate of complications and poor survival despite normalization of gas exchange within 48 h. Presenting one of the largest cohorts of patients requiring this kind of support, data collected from PELS‐1 database show how these patients had a high prevalence of comorbidities and underwent complex and long surgeries, with an impact on postoperative complications and poor outcomes.

High mortality rate and extensive resource utilization call for some careful evaluations before V‐V ECMO initiation in cardiac surgery patients. Timing is crucial when extracorporeal respiratory support is required, especially in fragile populations, and late initiation of ECMO may jeopardize outcomes. Early recognition of isolated respiratory failure, careful exclusion of significant cardiac dysfunction, and prompt initiation of ECMO may contribute to improving outcomes. Indications to V‐V ECMO after cardiac surgery should be tailored, taking into account medical history, institutional experience, and targets of care.

## Author Contributions

Concept/design: P.N., S.M., M.E.D.P., R.L. Data analysis/interpretation: B.C.T.B., A.‐K.S., M.P., U.B., K.B., J.J.H.B., H.B., B.M., M.L.S.M., K.R., F.F., P.S., D.C., R.D., J.‐S.J., J.B., G.B., A.B., K.S., G.W. Statistics: P.N., S.M., M.E.D.P. Data collection: M.D.M., D.S., L.B., R.S., X.H., L.S., M.A.M., S.S., C.R., A.F., G.M.R., I.‐W.W., V.P., M.P., J.P.G. Drafting article: P.N., S.M., M.E.D.P., R.L. Critical revision of article: B.C.T.B., M.D.M., A.‐K.S., D.S., M.P., L.B., U.B., R.S., K.B., X.H., J.J.H.B., H.B., L.S., B.M., M.A.M., M.L.S.M., S.S., K.R., C.R., F.F., P.S., A.F., D.C., G.M.R., R.D., I.W.W., J.‐S.J., J.B., V.P., G.B., M.P., A.B., J.P.G., K.S., G.W. Approval of article: all authors (P.N., S.M., M.E.D.P., B.C.T.B., M.D.M., A.‐K.S., D.S., M.P., L.B., U.B., R.S., K.B., X.H., J.J.H.B., H.B., L.S., B.M., M.A.M., M.L.S.M., S.S., K.R., C.R., F.F., P.S., A.F., D.C., G.M.R., R.D., I.W.W., J.‐S.J., J.B., V.P., G.B., M.P., A.B., J.P.G., K.S., G.W., R.L.).

## Conflicts of Interest

P.N.: honorarium from Abiomed for educational lectures; R.L.: consultant for Medtronic‐Abiomed‐LivaNova; Speaker for Abiomed; Advisory Board Member of Eurosets‐Hemocue‐Xenios (honoraria as research funding). D.W.: Consultat/Proctor for Abbott; Scientific advisor for Xenios.

## Supporting information


**Data S1:** aor70093‐sup‐0001‐Supinfo.docx.

## Data Availability

The data that support the findings of this study are available from the corresponding author upon reasonable request.
