# Supplementary material for: Outcomes in Patients Requiring Veno‐Venous Extracorporeal Membrane Oxygenation After Cardiac Surgery: An Analysis From the PELS‐1 Study
Source: Artif Organs. 2026 Jan 11;50(5):745–54. doi: 10.1111/aor.70093 (PMC13206365; doi:10.1111/aor.70093)
Supplement: Supplementary file 1 — Data S1: aor70093‐sup‐0001‐Supinfo.docx. [file AOR-50-745-s001.docx]

***Supplemental Digital Content***

OUTCOMES IN PATIENTS REQUIRING VENO-VENOUS EXTRACORPOREAL MEMBRANE OXYGENATION FOLLOWING CARDIAC SURGERY: AN ANALYSIS FROM THE PELS-1 MULTICENTER COHORT STUDY

*Table of contents:*

| Complete affiliations and list of PELS Investigators | Page 2 |
| --- | --- |
| Data collection | Page 6 |
| Variables definition | Page 8 |
| **Supplementary Figure 1**. Flowchart of V-V ECMO patients in PELS-1 Study | Page 12 |
| **Supplementary Figure 2**. Lactates Trend over time in patients requiring postcardiotomy V-V ECMO | Page 13 |
| **Supplementary Table 1.** Type of surgery and surgery characteristics of patients requiring V-V ECMO | Page 14 |

Complete affiliations and list of PELS Investigators

^1,2^Pasquale Nardelli; ^1,3^Silvia Mariani; ^1^Maria Elena De Piero; ^1,4,5^Bas C.T. van Bussel; ^6^Samuel Heuts; ^1^Michele Di Mauro; ^7,8^Dominik Wiedemann; ^7^Ann-Kristin Schaefer, ^7^Luca Conci ; ^9^Diyar Saeed; ^10^Jawad Khalil; ^10^Sven Lehmann; ^11^Matteo Pozzi; ^11^Jean-Francois Obadia; ^12^Luca Botta; ^12,13^Antonio Loforte; ^12^Davide Pacini; ^14^Udo Boeken; ^14^Nikolaos Kalampokas; ^15^Robertas Samalavicius; ^15^Agne Jankuviene; ^16^Karl Bounader; ^16^Erwan Flecher; ^17^Xiaotong Hou; ^18,19^Jeroen J.H. Bunge; ^18,19^Dinis Dos Reis Miranda; ^20^Hergen Buscher; ^20^Kogulan Sriranjan; ^21^Leonardo Salazar; ^22^Bart Meyns; ^23^Michael A Mazzeffi; ^23^Daniel Herr; ^24^Marco L Sacha Matteucci; ^24^Marco Di Eusanio; ^25^Sandro Sponga; ^25^Igor Vendramin; ^26^Graeme MacLaren; ^26^Vitaly Sorokin; ^26^Kollengode Ramanathan; ^27^Claudio Russo; ^27^Alessandro Costetti; ^28^Francesco Formica; ^29,30^Giovanni Marchetto; ^31^Pranya Sakiyalak; ^32^Antonio Fiore; ^33^Daniele Camboni; ^33^Chistof Schmid; ^34,35^Giuseppe Maria Raffa; ^36^Rodrigo Diaz; ^36^Roberto Castillo; ^37^I-wen Wang; ^38^Jae-Seung Jung; ^39^Jan Belohlavek; ^40^Tomas Grus; ^41^Vin Pellegrino; ^42^Giacomo Bianchi; ^42^Marco Solinas; ^43^Matteo Pettinari; ^44^Alessandro Barbone; ^45^José P. Garcia; ^46^Kiran Shekar; ^47^Glenn Whitman; ^1,48^Roberto Lorusso.

**Affiliations:**

1. Cardiovascular Research Institute Maastricht (CARIM), Maastricht University, Maastricht, Netherlands.
2. Cardiac Critical Care Unit, IRCCS San Raffaele Scientific Institute, Milan, Italy
3. Cardiac Surgery Unit, Fondazione IRCCS San Gerardo dei Tintori, Monza, Italy
4. Department of Intensive Care Medicine, Maastricht University Medical Center, Maastricht, The Netherlands
5. Care and Public Health Research Institute, Maastricht University, Maastricht, Netherlands
6. Cardio-Thoracic Surgery Department, Maastricht University Medical Centre, Maastricht, The Netherlands
7. Department of Cardiac Surgery, Medical University of Vienna, Vienna, Austria
8. Department of Cardiac Surgery, Karl Landsteiner University, University Clinic St. Pölten, St. Pölten, Austria
9. Heart Center Niederrhein, Helios Hospital Krefeld, Krefeld, Germany
10. Department of Cardiac Surgery, Leipzig Heart Center, Leipzig, Germany
11. Department of Cardiac Surgery, Louis Pradel Cardiologic Hospital, Lyon, France
12. Division of Cardiac Surgery, IRCCS Azienda Ospedaliero-Universitaria di Bologna, Bologna, Italy
13. University of Turin, Turin, Italy
14. Department of Cardiac Surgery, Medical Faculty, Heinrich Heine University, Duesseldorf, Germany
15. II Department of Anesthesiology, Centre of Anesthesia, Intensive Care and Pain management, Vilnius University Hospital Santariskiu Klinikos, Vilnius, Lithuania
16. Division of Cardiothoracic and Vascular Surgery, Pontchaillou University Hospital, Rennes, France
17. Center for Cardiac Intensive Care, Beijing Institute of Heart, Lung, and Blood Vessels Diseases, Beijing Anzhen Hospital, Capital Medical University, Beijing, China
18. Department of Intensive Care Adults, Erasmus MC, Rotterdam, The Netherlands
19. Deparment of Cardiology, Thoraxcenter, Erasmus MC, Rotterdam, The Netherlands
20. Department of Intensive Care Medicine, Center of Applied Medical Research, St Vincent's Hospital, Darlinghurs, NSW, and University of New South Wales, Sidney, Australia
21. Department of Cardiology, Fundación Cardiovascular de Colombia, Bucaramanga, Colombia
22. Department of Cardiovascular Sciences, University of Leuven, Leuven, Belgium
23. Departments of Medicine and Surgery, University of Maryland, Baltimore, USA
24. SOD Cardiochirurgia Ospedali Riuniti 'Umberto I - Lancisi - Salesi' Università Politecnica delle Marche, Ancona, Italy
25. Division of Cardiac Surgery, Cardiothoracic Department, University Hospital of Udine, Udine, Italy
26. Cardiothoracic Intensive Care Unit, National University Heart Centre, National University Hospital, Singapore, Singapore
27. Cardiac Surgery Unit, Cardiac Thoracic and Vascular Department, Niguarda Hospital, Milan, Italy
28. Cardiac Surgery Clinic, University of Salento, Department of Experimental Medicine, Vito Fazzi Hospital, Lecce, Italy
29. Cardiac Surgery Unit, Fondazione IRCCS San Gerardo dei Tintori, Monza, Italy
30. University of Milan-Bicocca, Milan, Italy
31. Division of Cardiovascular and Thoracic Surgery, Department of Surgery, Faculty of Medicine Siriraj Hospital, Mahidol University, Bangkok, Thailand
32. Department of Cardio-Thoracic Surgery, University Hospital Henri-Mondor, Créteil, Paris, France
33. Department of Cardiothoracic Surgery, University Medical Center Regensburg, Regensburg, Germany
34. Department for the Treatment and Study of Cardiothoracic Diseases and Cardiothoracic Transplantation, IRCCS-ISMETT (Istituto Mediterraneo per i Trapianti e Terapie ad Alta Specializzazione), Palermo, Italy
35. Cardiac Surgery Unit, Department of Precision Medicine in Medical Surgical and Critical Area (Me.Pre.C.C.), University of Palermo, 90134 Palermo, Italy.
36. ECMO Unit, Departamento de Anestesia, Clínica Las Condes, Las Condes, Santiago, Chile
37. Division of Cardiac Surgery, Memorial Healthcare System, Hollywood, FL 33021, USA
38. Department of Thoracic and Cardiovascular Surgery, Korea University Anam Hospital, Seoul, South Korea
39. 2nd Department of Internal Medicine, Cardiovascular Medicine General Teaching Hospital and 1st Faculty of Medicine, Charles University in Prague, Prague, Czech Republic
40. 2nd Department of Cardiovascular Surgery, First Faculty of Medicine, Charles University and General University Hospital in Prague, Prague, Czech Republic
41. Intensive Care Unit, The Alfred Hospital, Melbourne, VIC, Australia
42. Ospedale del Cuore Fondazione Toscana "G. Monasterio", Massa, Italy
43. Department of Cardiovascular Surgery, Ziekenhuis Oost-Limburg, Genk, Belgium
44. Cardiac Surgery Unit, IRCCS Humanitas Research Hospital – Rozzano (MI) – Italy
45. IU Health Advanced Heart & Lung Care, Indiana University Methodist Hospital, Indianapolis, IN, USA
46. Adult Intensive Care Services, The Prince Charles Hospital, Brisbane, Australia
47. Cardiac Intensive Care Unit, Johns Hopkins Hospital, Baltimore, Maryland, USA
48. Cardio-Thoracic Surgery Department, Maastricht University Medical Centre, Maastricht, The Netherlands

**Data collection**

The following predefined groups of data were collected:

- Demographic data: sex, age, race
- Patients characteristics: EuroSCORE, length, weight, serum creatinine level, left ventricular ejection fraction, comorbidities (hypertension, chronic kidney disease requiring dialysis, previous myocardial infarction, previous endocarditis, smoking, previous stroke, atrial fibrillation, previous pulmonary embolism, diabetes mellitus, previous transient ischemic attack (TIA), implanted pacemaker (PM), implanted implantable cardioverter defibrillator (ICD), previous percutaneous coronary intervention (PCI), chronic obstructive pulmonary disease (COPD), peripheral artery disease, chronic pulmonary embolism, asthma, pulmonary hypertension, previous cardiac surgery, implanted left ventricular assist device (LVAD), New-York Heart Association class.
- Preoperative status: urgency of the procedure, weight of intervention, planned intervention, preoperative cardiogenic shock, preoperative intubation, preoperative cardiac arrest, preoperative septic shock, preoperative vasopressors, preoperative acute pulmonary oedema, preoperative intra-aortic balloon pump (IABP), preoperative right ventricular failure, preoperative biventricular failure.
- Diagnosis: coronary artery disease, aortic vessel disease, aortic valve disease, mitral valve disease, tricuspid valve disease, pulmonary valve disease, post-acute myocardial infarction (AMI) ventricular septal rupture, free wall/Papillary muscle rupture, graft failure, active endocarditis, atrial septal defect, post-LVAD right ventricular failure, other diagnosis
- Coronary surgery: arterial graft, number of distal arterial anastomoses, left internal mammary artery (LIMA), right internal mammary artery (RIMA), radial artery, gastro-epiploic artery (GEA), other arterial graft, venous graft, number of distal venous anastomoses, other coronary surgery
- Valve surgery: valve surgery, aortic valve surgery, aortic valve procedure, mitral valve surgery, mitral valve procedure, pulmonary valve surgery, pulmonary valve procedure, pulmonary valve implant, tricuspid valve surgery, tricuspid valve procedure.
- Aortic surgery: approach to aortic surgery, aortic ascending surgery, aortic arch surgery, descending aortic procedure.
- Other cardiac surgeries: cardiac assist device, heart transplantation, rhythm surgery, additional PM-/ICD procedure, ventricular septal defect (VSD) closure, atrial septal defect (ASD) closure, ventricular surgery, pericardiectomy, pulmonary embolectomy/endarterectomy, other cardiac surgery, other cardiac surgery description.
- Extracorporeal circulation (ECC): ECC duration, cross-clamp duration, circulation arrest, cardioplegia characteristics, off-pump conversion.
- Extracorporeal membrane oxygenation (ECMO) variables: ECMO indication, chest status, cannulation approach, use of left ventricular vent, ECMO duration (hours), configuration change, ECMO monitoring.
- In-hospital outcomes: deceased in hospital, deceased timing, intensive care unit stay (days), hospital stay (days), in-hospital mortality, death timing, postoperative bleeding (requiring re-thoracotomy, cannulation site bleeding, diffuse no-surgical related bleeding), neurological complications (brain oedema, cerebral haemorrhage, seizure, stroke, vasospasm), arrhythmia, leg ischemia, cardiac arrest, pacemaker implant, bowel ischemia, right ventricular failure, acute kidney injury, pneumonia, septic shock, distributive shock syndrome, acute respiratory distress syndrome (ARDS), multi-organ failure, embolism
- Postoperative procedures: PCI, new cardiac surgery, abdominal surgery, vascular surgery
- Outcomes at follow-up: mortality status, follow-up time

**Variable definitions**

The following definitions were used for the main study variables:

- Sex: our research and manuscript have been developed in accordance to the international World Health Organization definitions of sex and gender where ‘Gender’ describes those characteristics of women and men that are largely socially created (including concepts such as cisgender and transgender). while ‘sex’ encompasses those that are biologically determined (https://www.who.int/genomics/gender/en/). The same distinction is mirrored by the definitions of gender and sex given by several other international institutions such as the World Health Organization Regional Office for Europe (https://www.euro.who.int/en/health-topics/health-determinants/gender/gender-definitions), the Office for National Statistics and United Kingdom government (https://www.ons.gov.uk/economy/environmentalaccounts/articles/whatisthedifferencebetweensexandgender/2019-02-21) or the Canadian Institutes of Health Research (https://cihr-irsc.gc.ca/e/48642.html). Despite these definitions, sex and gender are often mistakenly used interchangeably in scientific literature, health policy, and legislation. In our study, we defined our patients based on biologically determined sex (male/female) and we did not include a further analysis of gender identity ^2-4^.
- Hypertension: Systolic blood pressure >140mmHg or diastolic blood pressure >90mmHg^5^, or use of antihypertensive agents to maintain normal blood pressure
- Smoking: active (smoking during the past 30 days) and more than 100 cigarettes during lifetime
- COPD: Diagnosis of chronic obstructive pulmonary disease, any Gold classification ^6^
- Peripheral arterial disease: Claudication, carotid occlusion or >50% stenosis, amputation for arterial disease or previous or planned intervention on the abdominal aorta. limb arteries or carotids ^7^
- Pulmonary hypertension: Systolic pulmonary artery pressure >50mmHg
- EuroSCORE II: European System for Cardiac Operative Risk Evaluation II proposing a risk assessment of cardiac surgical procedures which incorporates patient age, sex, diabetic status, pulmonary disease, neurological function, renal function, presence of active endocarditis, pre-operative state, procedural urgency and procedure type^7^
- New York heart association class: Functional class of dyspnoea according to the classification as proposed by the New York Heart Association
- Preoperative cardiogenic shock: Preoperative state with life-threatening hypotension despite rapidly escalating inotropic support. critical organ hypoperfusion, with worsening acidosis and/or lactate levels^8^
- Preoperative cardiac arrest: Preoperative cardiopulmonary resuscitation in the 24 hours prior to surgery
- Preoperative septic shock: Septic patients with vasopressor requirement to maintain mean arterial pressure >65mmHg and serum lactate levels greater than 2mmol/L in the absence of hypovolemia^9^
- Preoperative right ventricular failure: Evidence of right-sided structural and/or functional abnormalities in combination with clinical symptoms and signs of right ventricular failure^10^
- Preoperative biventricular failure: Biventricular dysfunction accompanied by both signs and symptoms of right-sided and left-sided heart failure^11^
- Emergency surgery: Surgery before the beginning of the next working day after the decision to operate is made^7^
- Urgent surgery: Patients not electively admitted for operation but requiring surgery during the current admission without a possibility to be discharged before undergoing the definite procedure ^7^
- Aortic vessel disease: Any disease of the ascending aorta. aortic arch or proximal descending aorta warranting surgical correction during the current procedure
- Aortic valve disease: Any aortic valve disease, including (prosthetic) aortic valve stenosis, regurgitation and endocarditis
- Mitral valve disease: Any mitral valve disease, including (prosthetic) mitral valve stenosis, regurgitation and endocarditis
- Tricuspid valve disease: Any tricuspid valve disease, including (prosthetic) tricuspid valve stenosis. regurgitation and endocarditis
- Pulmonary valve disease: Any pulmonary valve disease, including (prosthetic) pulmonary valve stenosis, regurgitation and endocarditis
- Active endocarditis: Patients still on antibiotic treatment for endocarditis at the time of surgery^7^
- Post LVAD right ventricular failure: Right ventricular failure as described previously in presence of LVAD
- Ventricular surgery: Surgery performed to restore structural ventricular function, especially in case of ventricular aneurysm formation or rupture
- Rhythm surgery: Surgical (either epicardial or endo-epicardial) ablation performed for atrial or ventricular arrhythmia
- Failure to wean: Failure to wean from cardio-pulmonary bypass despite preload optimization and completeness of surgery
- Arrhythmia: Refractory ventricular arrhythmia with uncontrollable hemodynamic consequences
- Cardiac arrest: Abrupt loss of heart function despite acute and simple interventions such as pacing and defibrillation
- Cardiogenic shock: State of life-threatening hypotension despite rapidly escalating inotropic support, critical organ hypoperfusion, with worsening acidosis and/or lactate levels ^8^
- Right ventricular failure: Evidence of right-sided structural and/or functional abnormalities in combination with clinical symptoms and signs of right ventricular failure ^10^
- Respiratory failure: Reversible pulmonary disease which cannot anymore be managed by conventional mechanical ventilation, despite optimization of pharmacological interventions with or without prone positioning
- Biventricular failure: Biventricular dysfunction accompanied by both signs and symptoms of right-sided and left-sided heart failure ^11^
- Chest closed: Any cannulation condition in which the sternum is closed irrespective location of cannulas
- Chest open: Any cannulation condition in which the sternum is left open irrespective of skin closure
- Stroke: Neurological dysfunction caused by focal brain or retinal ischemia with clinical symptoms lasting less more than 24 hours, with or without permanent disability
- Transient ischemic attack: A brief episode of neurological dysfunction caused by focal brain or retinal ischemia with clinical symptoms lasting less than one hour, without evidence of acute brain infarction ^12^
- Arrhythmia: Any atrial or ventricular arrhythmia lasting more than 30 seconds
- Leg ischemia: Clinical signs of lower extremity ischemia requiring intervention (either by vascular surgery or cannula removal)
- Bowel ischemia: Intestinal ischemia with elevated lactate levels requiring abdominal surgical intervention
- Acute kidney injury: Postoperative requirement for dialysis while not on dialysis before or duplication of preoperative creatinine levels (and absolute creatinine level >177μmol/L)
- Pneumonia: Any (suspected) pulmonary infection treated with antibiotics
- Septic shock: Sepsis with vasopressor requirement to maintain mean arterial pressure >65mmHg and serum lactate levels greater than 2mmol/L in the absence of hypovolemia^9^
- Distributive shock syndrome: mean arterial pressure <50mmHg with cardiac index >2,5L/min/m^2^, right atrial pressure <5mmHg, left atrial pressure <10mmHg an low systemic vascular resistance (<800 dyne/s/cm^-5^) during intravenous norepinephrine infusion (>0,5μg/kg/min)^13^
- ARDS: Acute diffuse inflammatory lung injury requiring invasive mechanical ventilation of extracorporeal membrane oxygenation
- Multi-organ failure: Hypometabolic state with involvement of more than one organ as established by biochemical and/or radiological analysis.

**Supplementary Figure 1**. Flowchart of V-V ECMO patients in PELS-1 Study


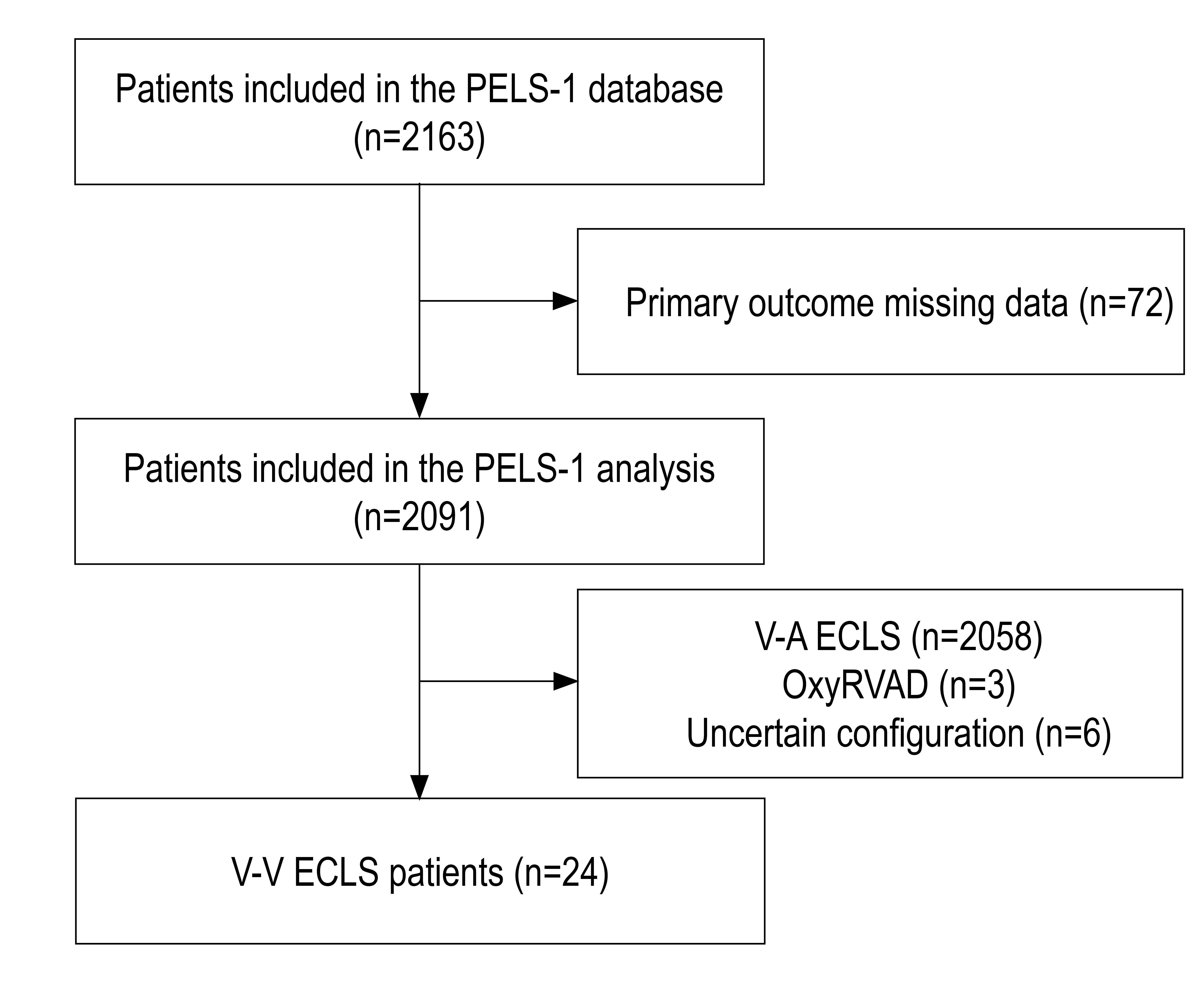


**Supplementary Figure 2**. Lactates Trend over time in patients requiring postcardiotomy V-V ECMO


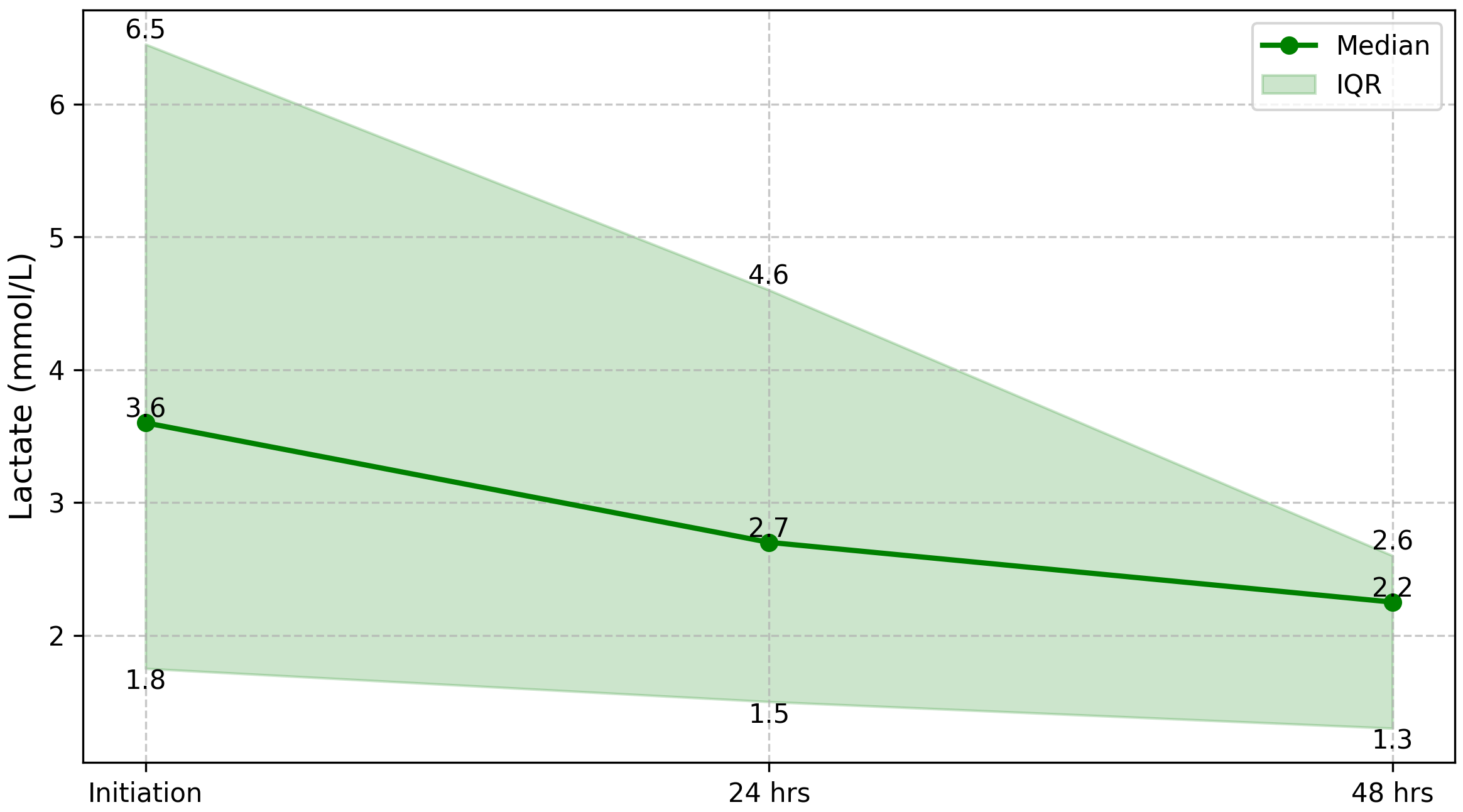


**Supplementary Table 1.** Type of surgery and surgery characteristics of patients requiring V-V ECMO

|  | **V-V ECMO patients**  *n=24* | *Data availability* |
| --- | --- | --- |
| ***Type of surgery*** | | |
| Coronary artery bypass | 3 (12.5%) | *24 (100%)* |
| Aortic valve surgery | 13 (54.2%) | *24 (100%)* |
| Mitral valve repair | 3 (12.5%) | *24 (100%)* |
| Mitral valve replacement | 4 (16.7%) | *24 (100%)* |
| Tricuspid valve repair | 3 (12.5%) | *24 (100%)* |
| Ascending aorta and root replacement | 5 (20.8%) | *24 (100%)* |
| Ascending aorta replacement | 3 (12.5%) | *24 (100%)* |
| Ascending aorta and arch replacement | 3 (12.5%) | *24 (100%)* |
| Heart transplantation | 1 (4.2%) | *24 (100%)* |
| Weight of surgery | |  |
| Isolated CABG | 1 (4.2%) | *24 (100%)* |
| Isolated non-CABG | 18 (75%) | *24 (100%)* |
| 2 procedures | 1 (4.2%) | *24 (100%)* |
| 3 or more procedures | 4 (16.7%) | *24 (100%)* |
| ***Surgery characteristics*** | | |
| CPB used | 23 (100%) | *23 (96%)* |
| Crossclamp time (min) | 114 [56–200] | *23 (96%)* |
| CPB time (min) | 208 [110–350] | *23 (96%)* |
| Multiple CPB runs | 0 | *23 (96%)* |
| Cardioplegia type | | *20 (83%)* |
| Blood | 6 (30%) |  |
| Crystalloid | 7 (35%) |  |
| Custodiol | 6 (30%) |  |
| Other | 1 (5%) |  |
| Intraoperative transfusions | 16 (100%) | *16 (67%)* |
| Postoperative IABP | 2 (12.5%) | *24 (100%)* |
| *Continuous variables are presented as median with 1st and 3rd quartile. Categorical variables are expressed as frequencies and percentages.*  *Abbreviations: CABG (coronary artery bypass graft), CPB (cardiopulmonary bypass), IABP (intra-aortic balloon pump).* | | |
